# Temperature dependence of electrical conductivity and variable hopping range mechanism on graphene oxide films

**DOI:** 10.1038/s41598-023-31778-3

**Published:** 2023-03-23

**Authors:** D. J. Sánchez-Trujillo, L. V. Osorio-Maldonado, J. J. Prías-Barragán

**Affiliations:** 1 Electronic Engineering Program, Faculty of Engineering at Universidad del Quindío, 630004 Armenia, Colombia; 2 Doctoral Program in Physical Sciences, Interdisciplinary Institute of Sciences, Electronic Instrumentation Technology Program, Faculty of Basic Sciences and Technology at Universidad del Quindío, 630004 Armenia, Colombia

**Keywords:** Materials science, Optics and photonics

## Abstract

The rapid development of optoelectronic applications for optical-to-electrical conversion has increased the interest in graphene oxide material. Here, graphene oxide films (GOF) were used as source material in an infrared photodetector configuration and the temperature dependence of the electrical conductivity was studied. GOF were prepared by the double-thermal decomposition (DTD) method at 973 K, with a fixed carbonization temperature, in a pyrolysis system, under a controlled nitrogen atmosphere, over quartz substrates. Graphene oxide films were mechanically supported in a photodetector configuration on Bakelite substrates and electrically contacted with copper wires and high-purity silver paint. Morphological images from the GOF’s surface were taken employing a scanning electron microscope and observed a homogeneous surface which favored the electrical contacts deposition. Vibrational characteristics were studied employing Raman spectroscopy and determined the typical graphene oxide bands. GOF were used to discuss the effect of temperature on the film’s electrical conductivity. Current–voltage (I–V) curves were taken for several temperatures varying from 20 to 300 K and the electrical resistance values were obtained from 142.86 to 2.14 kΩ. The GOF electrical conductivity and bandgap energy (E_g_) were calculated, and it was found that when increasing temperature, the electrical conductivity increased from 30.33 to 2023.97 S/m, similar to a semiconductor material, and E_g_ shows a nonlinear change from 0.33 to 0.12 eV, with the increasing temperature. Conduction mechanism was described mainly by three-dimensional variable range hopping (3D VRH). Additionally, measurements of voltage and electrical resistance, as a function of wavelength were considered, for a spectral range between 1300 and 3000 nm. It was evidenced that as the wavelength becomes longer, a greater number of free electrons are generated, which contributes to the electrical current. The external quantum efficiency (EQE) was determined for this proposed photodetector prototype, obtaining a value of 40%, similar to those reported for commercial semiconductor photodetectors. This study provides a groundwork for further development of graphene oxide films with high conductivity in large-scale preparation.

## Introduction

In recent years, graphene (G) and their oxide derivatives, graphene oxide (GO) and reduced graphene oxide (rGO), have attracted great interest in basic research and technological applications^1–4^, given their exceptional physical properties. It is well known that due to graphene oxidation, there are physical and chemical differences between G, rGO and GO^5,6^; for example, they exhibit high, medium, and low optical transparency, electrical and thermal conduction, respectively. These physical and chemical properties are modified due to the presence of oxides out of the carbon atom plane which produces structural disorder ^7–9^, and hence, electrical conductivity can reach in some cases higher values than 2000^10,11^, 1250^12^, and 71^13^ S/m, respectively. The considerable amount of structures derived from rapid progress in the fabrication and transfer techniques of graphene and its remarkably distinct electronic properties^14,15^, have been utilized in a wide range of electronic sensors and devices based on these materials, such as radio frequency (RF) filters^16^, infrared (IR) photodetectors^17–20^, phototransistor with colloidal quantum dots^21^, light emitting diodes (LEDs)^22–25^ and field-effect transistors (FETs)^26–28^, among many others^29^. Recent advances on the topic, suggest the characterizations of electrical, optical, and thermal properties in graphene oxide films (GOF)^7–9^ is an interesting open research field.

Due to light-matter interactions, photodetectors based on rGO have received great interest in basic and in several technological applications. In particular, broadband photodetectors^17^, IR photodetectors^30,31^ and plasmon-enhanced photodetectors^32^ have been developed. Recently, the photoresponse properties of rGO/n-silicon^33^ have been studied, allowing rGO to be considered one of the best candidates for IR photodetection, due to its chemical stability and electrical response. However, to improve infrared photodetectors, it is required high surface quality and high external quantum efficiency (EQE), as proposed here.

Given this technological potential, electronic properties like the conductivity and carrier mobility have been studied in single and multilayered graphene and graphene oxide from the experimental point of view ^10,13,14,34,35^ and computational simulations^36^. Yan et al.^14^ investigated the oxidation effect on the structural and electronic properties of graphene based on first-principles calculations, finding that a band gap ranging from 0.1 to 4.0 eV can be obtained by changing the oxidation level and the location of the oxidized region. Murata et al.^10^ synthesized uniform multilayered graphene layers of various thicknesses, varying from 5 to 200 nm, which exhibited electrical conductivity around 2,700 S/cm, and which exceeded the conductivity in highly oriented pyrolytic graphite. Chen Jing et al.^37^ developed two-stacked graphene monolayers with a clean interface, finding that by studying the resistance vs gate voltage curves, the electronic transport properties of this material are quite different from monolayered and bi-layered graphene. Also, Chen et al.^13^ presented nano-fibrillated cellulose and GO composite films to develop conductive paper-like films, with electrical conductivities ranging from 22.2 to 168.9 S/m.

Many of these studies agree that the main electrical transport mechanism involved in GO samples is the variable-range hopping mechanism^34,38–44^. Young et al.^38^ investigated the low-temperature electron transport properties of rGO sheets, with different carbon sp^2^ fractions from 55 to 80% and showed that in the low-bias (Ohmic) regime, the temperature-dependent resistance of all the devices followed the Efros-Shklovskii (ES) variable-range hopping. Govind Raj et al.^44^ studied changes in electrical transport properties and the conduction mechanism in disordered carbon, with the extent of graphitization, finding that the conduction mechanism is modified from the 3-dimensional variable-range hopping (3D VRH) model, to the 2-dimensional weak localization (2D WL) model. Eda et al.^43^ presented the transport properties of progressively reduced GO showing that, the evolution of electronic properties reveals that GO undergoes insulator-semiconductor-semimetal transitions with reduction and that transport in reduced GO occurs via VRH and further reduction leads to an increased number of available hopping sites.

In addition, the temperature dependence of the electrical conductivity has been considered in some cases, such as the work by Muchharla et al.^45^ in which temperature-dependent electrical transport properties of rGO thin films have been studied in a wide range (50 K < T < 400 K) of temperatures. Electrical conduction in rGO thin films was displayed in two different temperature regimes; at higher temperatures, an Arrhenius-like temperature dependent resistance was observed, indicating bandgap energy involved in electrical transport; at lower temperatures, the rGO sample showed a conduction mechanism consistent with Mottʼs two-dimensional variable range hopping (2D-VRH). Electrical measurements were performed mostly by employing the differential 4-probe technique, where 2 MΩ electrical resistance values have been measured, as presented by Jung et al.^41^. Other measurements were performed by employing two-point probes. Venugopal et al.^15^ obtained I-V curves in multilayered graphene field-effect devices and found Ohmic behavior at room temperature; also, Gross et al.^46^ introduced the oxygen content dependence of the electrical conductivity in GO single nanoplatelets obtained from bamboo pyroligneous acid (BPA), revealing that this GO material exhibited electrical transport like a narrow bandgap semiconductor.

Prías-Barragán et al.^47–49^ reported a new pyrolysis method for GO single nanoplatelets and GOF synthesis from BPA with efficiencies around 90%, as well as their studies on the structural, morphological, electrical, thermal, optical, and magnetic properties^48,50–52^, in addition to some potential applications, such as an IR emitter^47^, supercapacitors^53^, and optoelectronic devices^48^, among others. It has been identified that pyrolytic GO is a material with a polycrystalline structure, with magnetic behavior in ferromagnetism order at room temperature induced by topographic defects^54^, vibrational response of thermal insulator material (due to hydroxyl bridges)^50^, and whose multifunctional oxides give it a semiconductor electrical behavior with a narrow bandgap (0.11–0.30 eV)^46^. However, the temperature dependence of the electrical conductivity in GOF obtained from BPA remains unclear for this material. Here, we present the temperature dependence of electrical conductivity and 3D-VRH mechanism in GOF for potential applications in optoelectronic devices, such as the infrared photodetectors proposed here. The photodetector prototypes have been made from environmentally sustainable materials, taking advantage of the waste from the bamboo industry. It has been possible to understand the mechanism that governs photoelectron transport once they have been excited in photodetector configurations.

Therefore, the novelty highlighted in this work is that high surface quality GOF were used as source material in an infrared photodetector configuration with high EQE. In this regard, previous work has demonstrated that GOF are an excellent candidate material to development to IR detectors^7–9,55,56^. However, the electrical conduction mechanism in a pyrolytic GOF IR photodetector configuration, has not been yet studied at low temperatures, as discussed here, from the analysis of the temperature dependence of the electrical conductivity.

## Methods

### Sample preparation

**Figure 1:**
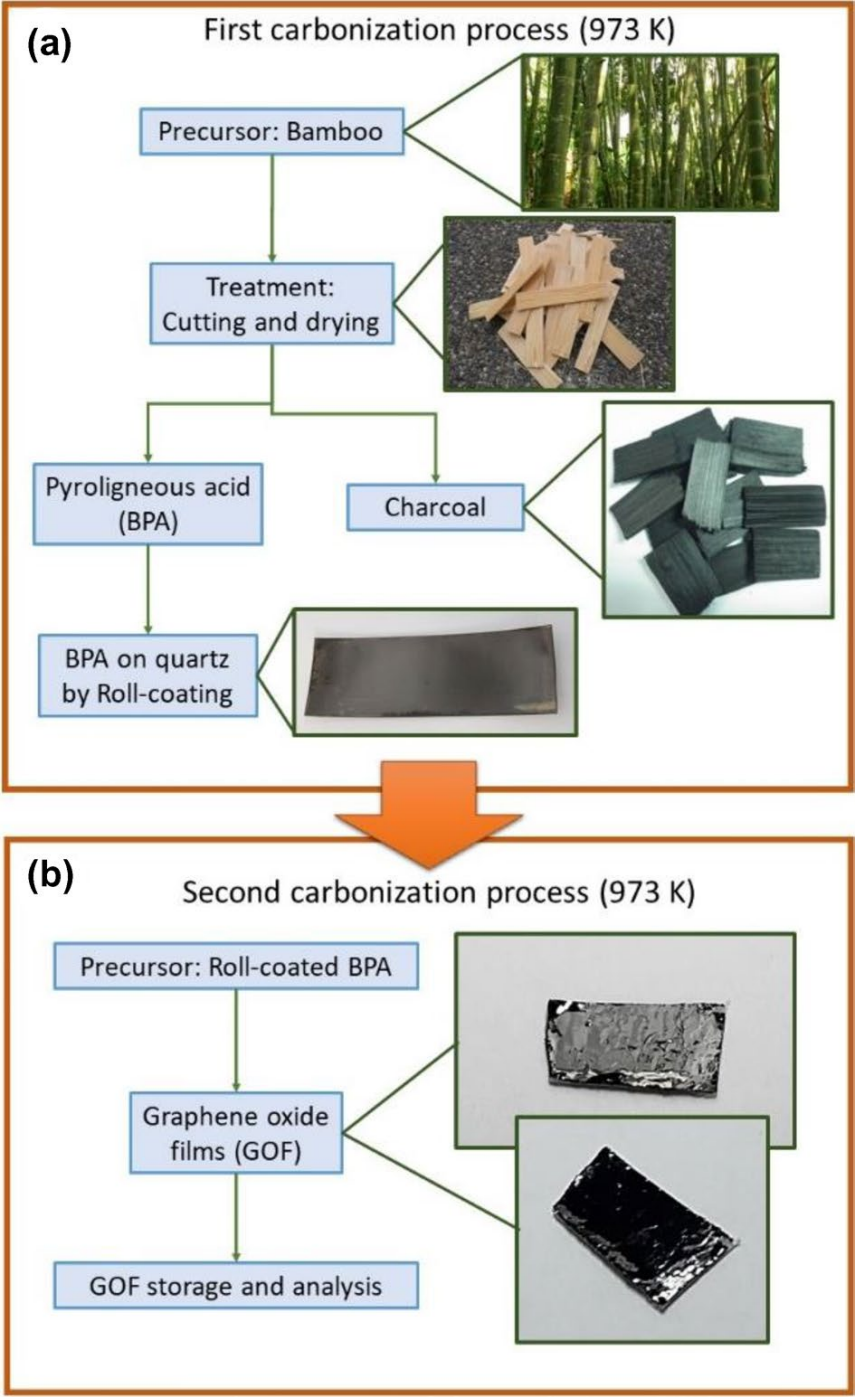
Two simple stages to obtain GOF. (**a**) First carbonization process where BPA is obtained, (**b**) second carbonization process where GOF are obtained. All the images presented as insets are the results of each step.

GO samples were obtained through the DTD method in a pyrolysis system under controlled nitrogen flux by employing pyroligneous acid from bamboo (Guadua *angustifolia Kunth*) as source material and reported before^47^. The DTD method involves two-step pyrolysis processes, as shown in Fig. 1: in the first pyrolysis step, from the carbonization process of the Bamboo raw material at 973 K for 1 h, BPA was obtained and collected in a decanting funnel glass; then, the BPA was deposited on quartz substrate by roll-coating. In the second pyrolysis step, roll-coated BPA was used as a source to obtain GOF at a fixed carbonization temperature of 973 K with an oxide atomic concentration of 5%, according to previous reports^46,48,49,52^. Then, GOF were obtained by a mechanical transfer method onto a Bakelite as substrate. For more details, see the references^47,48,51^. The authors confirm that all the methods in experimental research and field studies on plants, as the waste product of the commercial bamboo-guadua angustifolia Kunth, were performed in accordance with the relevant regulations.

### Device configuration

**Figure 2:**
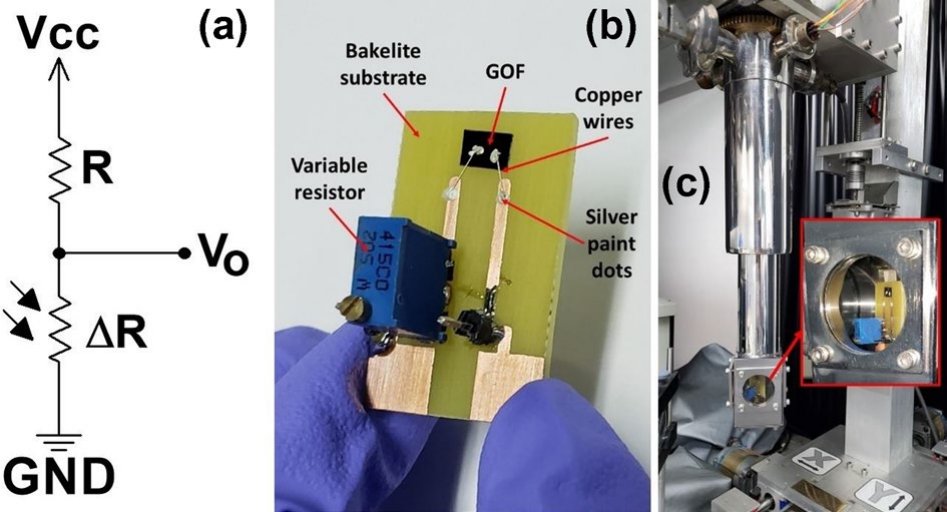
(**a**) Voltage divider configuration. (**b**) Photodetector image. (**c**) Photograph of the GOF photodetector prototype inside the cryostat system for low temperatures measurements.

Initially, the GOF were easily mechanically transferred from the quartz substrate employing a double-sided tape, given that they are attached by weak electrostatic forces. Then, it is cut into a 5 × 3 mm rectangle shape with a thin blade and, subsequently, each sample is adhered to the Bakelite substrate, on which a printed circuit (named the sample holder) is arranged to connect the electrical contacts of the photodetector. The thicknesses of the samples were measured, and the average value was around 6 µm. Finally, GOF were electrically contacted by employing high-purity silver paint spots and copper wires with 80 µm approximately of thicknesses, as shown in Figs. 2a and b. Photodetector prototypes were arranged by employing a voltage divider configuration for the electrical circuit (Fig. 2a). To allow a greater transfer of electrical power, in this configuration, an electrical resistance (1 kΩ) in series to the GOF photodetector was used (because this value is close to the electrical resistance measured in each GOF between 1.045 and 1.458 kΩ). To increase the signal-to-noise ratio of the measurements at room temperature, the photodetector prototype was located inside a black box (with an input for the monochromatic light beam), designed to prevent external light from several sources.

### Characterization methods

Morphological characterizations were carried out, employing Zeiss Evo 10 SEM equipment, with an accelerating voltage (EHT) of 20 kV, a working distance (WD) of 8.5 mm and a magnification of 2.5 kX. A Panalytical Systems diffractometer, Cu K_α_ radiation (a = 1.542 Å) was used to obtain X-ray diffraction patterns. For the comparative analysis, commercial graphite of analytical grade was used as reference in this work. Raman measurements were carried out at room temperature by using a confocal Horiba Jobin Yvon spectrometer, model Labram HR, with an excitation HeCd laser beam at 632.8 nm and an optical power of 0.25 mW.

For the electrical characterization, all GOF photodetector prototypes elaborated in this work were electrically characterized at different temperatures, from 20 to 300 K. A closed-cycle helium cooling system was used, incorporating the photodetector inside the cryostat, as shown in Fig. 2c. The pressure inside the cryostat was measured as the vacuum pump worked, employing a 917 Pirani sensor, while temperature was measured by using a Lake Shore 330 Autotuning temperature controller. I–V curves were obtained employing a Keithley 6220 precision current source and a Keithley 2182A precision nanovoltmeter, with resolutions of 100 fA and 1 nV, respectively. The current–voltage curves at different temperatures were obtained by connecting the photodetector prototype to the current source and the nanovoltmeter inside the cryostat system without the black box (Fig. 2c). The electrical current supplied to the photodetector prototype varied from −1 to 1 mA and the voltage was measured at the two electrical contacts device. The I–V curves were taken at different temperatures, varying from 20 to 300 K with an interval of 20 K. Electrical resistance and conductivity values were determined from the data analysis and the respective curves were plotted as a function of temperature for the range measured. In addition, the temperature dependence of these parameters in the proposed GOF was described by using the theoretical fitting to the experimental data, employing Mott's VRH model for the three-dimensional case, given by^38,57,58^:

1 $$\sigma ={\sigma }_{0}exp{\left(\frac{-{T}_{0}}{T}\right)}^{\frac{1}{d+1}}.$$

where σ_0_ is the conductance independent of temperature, *T*_0_ is the characteristic temperature, and *d* is the dimensionality of the system under investigation, here, the best fit was obtained employing *d* = 3. At low temperature this model can possibly describe the electrical conductivity response in GO samples. Additionally, in a diffusion hopping conduction process, the relation between the bandgap energy E_g_ and electrical conductivity it is often written as^59^:

2 $$\sigma \left(T\right)={\sigma }_{0}exp\left(-\frac{{E}_{g}}{2{k}_{B}T}\right),$$

here, *k*_*B*_*T* is the product of the Boltzmann constant, *k*_*B*_, and the temperature, *T*.

For the optical characterization, the wavelength influence on voltage and resistance was studied at room temperature, employing the following configuration: light from the quartz-tungsten-halogen (QTH) lamp operating at 120 W of electrical power was dispersed using a Triax 320 monochromator. The monochromatic light beam illuminated the GOF photodetector located inside the black box. The wavelength of the incident beam is considered in a range between 1300 and 3000 nm (near infrared). The electrical output is connected to a precision Keithley 197A millivoltmeter with a resolution of 100 μV, with which voltage and electrical resistance are measured with and without the incident beam, and values are stored. The influence of the wavelength on the voltage and electrical resistance were obtained. The photocurrent and the dark current are obtained employing Ohm's law. Then, the responsivity, *R,* and quantum efficiency, EQE*,* were calculated for each GOF from the following equations, respectively^60^:

3 $$R\left(\lambda \right)=\frac{{I}_{ph}}{{P}_{o}},$$

here, *I*_*ph*_ is the photocurrent generated and *P*_*o*_ is the incident optical power.

4 $$EQE=R\frac{e\lambda }{hc}g,$$

where, *h* is the Planck constant, *λ* is the wavelength and *g* is the gain, defined as the number of carriers detected per generated electron–hole pair, and indicates the performance of the collection system of photogenerated carriers.

## Results and discussion

### Morphological, vibrational and structural characterizations

**Figure 3:**
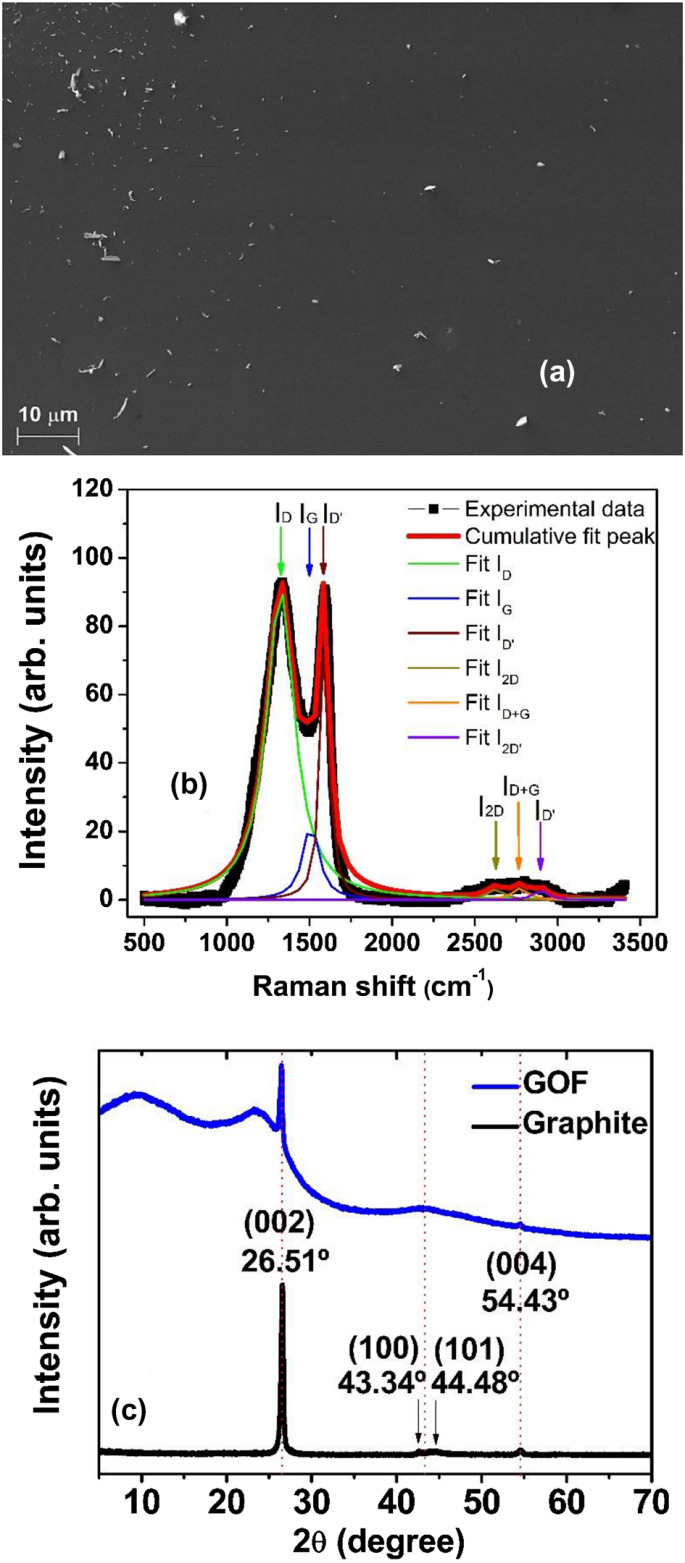
(**a**) SEM image of the GOF’s surface with high contrast by dispersed particles; (**b**) Raman spectra of GO sample obtained at room temperature and the respective fitting employing six Lorentzian peaks; (**c**) XRD in GO sample and in graphite sample, employed as reference.

Figure 3a shows a SEM image of a typical GOF’s surface obtained in the final stage of the process presented in Fig. 1. The homogeneity of the sample surface is highlighted here. White powder over the surface was used to improve the contrast image due to the high homogeneity of the GOF’s surface. The high homogeneity observed here favors the electrical contact deposition and optical response in GOF infrared photodetectors. The Raman spectra of a GO sample synthesized at 973 K is shown in Fig. 3b and the graphitic characteristic of GO is confirmed by this technique. Spectra were acquired in a range from 500 to 3400 cm^-1^. Figure 3b demonstrates the Raman spectra analysis carried out by using a fit with six Lorentzian function contributions associated to: D-band peak around 1325 cm^1^ that corresponds to the disorder-induced phonon mode by defects, related with the elastic scattering due to structural defects (grain boundaries, oxides, and sp^3^ defects); G-band peak around 1550 cm^1^ that indicates the formation of the graphitized structure by the vibration of sp^2^ bonded carbon atoms; D’ band peak at 1592 cm^-1^ associated to the Raman inelastic scattering due to the absorption or emission of phonons confined in defects, resulting in the expansion and contraction of GO layers; 2D, D + G, and 2D’ band peaks around 2625, 2790, and 2920 cm^-1^ respectively, suggests the presence of many stacked graphene oxide layers with edges, defects, and sp^2^ regions, as reported before^51^. The XRD pattern of GO synthesized at 973 K exhibits the polycrystalline structure and Bragg peaks assigned to both, graphite and GO materials. Slight differences among XRD peaks identified in Fig. 3c can be attributed to the presence of some oxides and intercalated compounds between graphene layers, which modify the layered film and the bonds separations in GOF samples^47^.

### Electrical characterization

**Figure 4:**
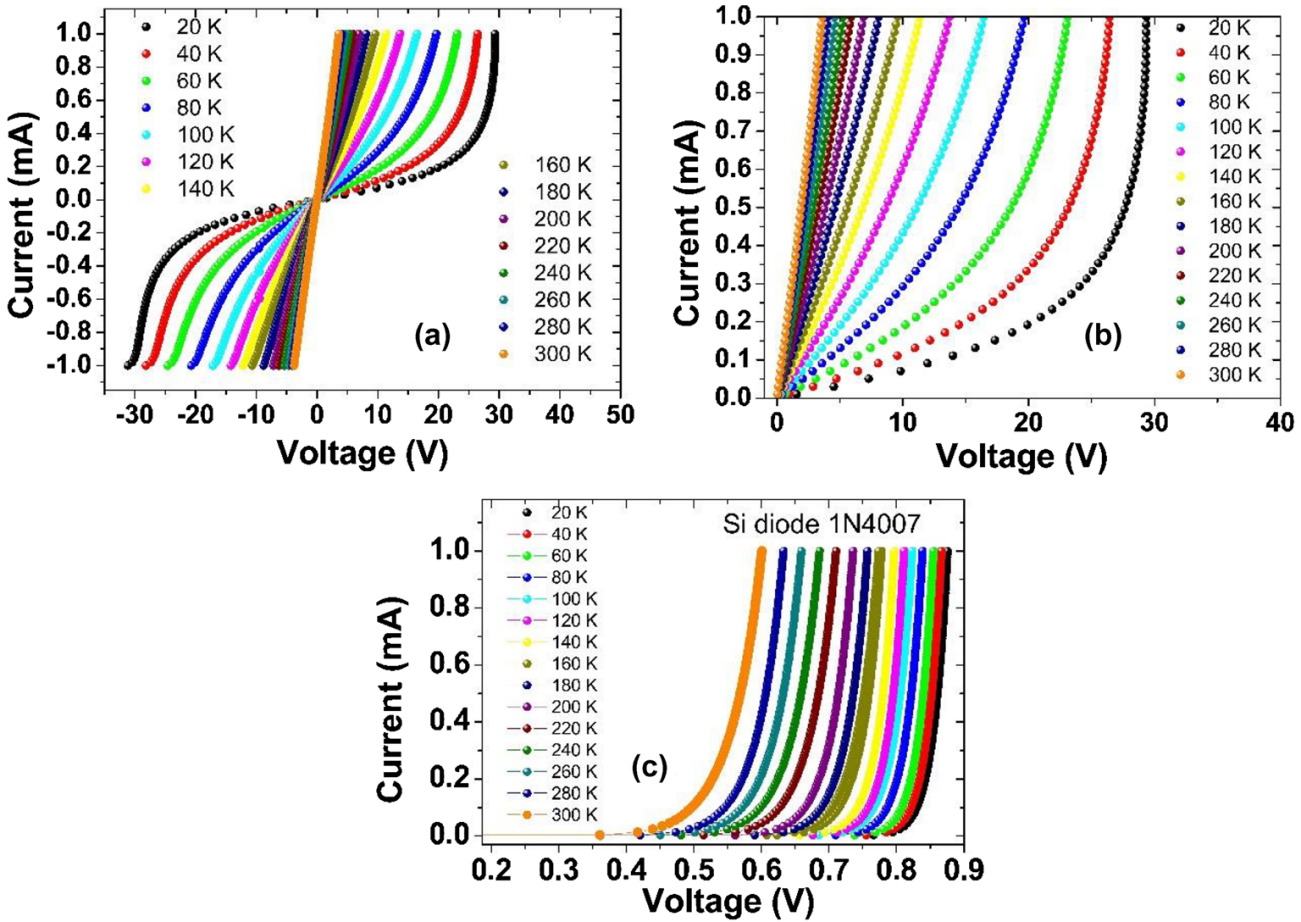
Effect of temperature on the current–voltage curves of one photodetector prototype based on GOF. (**a**) I–V curves taken from − 1 to 1 mA, (**b**) An approach in I–V curves in the positive range of current, (**c**) I–V curves for the Si diode 1N4007 employed as reference.

Figures 4a and b show I–V curves for one of the GOF photodetector prototypes at a temperature range between 20 and 300 K, considering both positive and negative electrical current values (a) and the approach to the positive ones (b). Figure 4c presents the measurements obtained for the 1N4007 silicon diode, used here as reference. It was observed that the I–V curve in all cases is temperature-dependent. For the commercial diode, as temperature increases, the barrier voltage decreases, as expected in devices with p-n junctions based on semiconductors^61,62^. In the prototypes fabricated in this work, a non-linear effect was observed in the I–V curves for low temperatures up to approximately 140 K, which is consistent with previous reports by Voitsihovska et al*.*^63^, and Rahaman et al.^64^ Also, for higher temperatures until 300 K, this effect disappears and an ohmic behavior is evidenced, within the foregoing range of currents, this behavior agrees with electrical linear responses for temperatures between 150 and 300 K, as reported by Abid et al.^65^, Bonavolontà et al.^17^, and Joung and Khondaker^38^ in rGO samples. This can be explained considering that, for low temperatures, the effect of the metal–semiconductor junction between the electrical contacts and the GOF sample becomes predominant. Moreover, carrier transport could be associated at these temperatures with the thermionic effect, as occurs in the commercial reference diode; or to the percolative effect of electrical current. It is well known that at low temperatures, phonon population decreases and hence, it reduces the effects of thermal noise in IR photodetectors, which enhance the main physical transport mechanisms. Therefore, to study the main electrical transport mechanism in GOF IR photodetectors, the measurements of I–V curves were taken at low temperatures with a minimum thermal noise. For temperatures above 150 K, dispersive processes occur in the material and domain the charge carrier transport, evidenced in the ohmic behavior, which can be attributed to that the increase of temperature produces a low variation of the bending of energy bands (HOMO and LUMO) that reduces the barrier voltage at the metal–semiconductor junction. Therefore, in the temperature range of 150 K to 300 K, the coupling between silver paint and GOF exhibit an ohmic response based on a dispersive transport mechanism, as reported before^65^.

**Figure 5:**
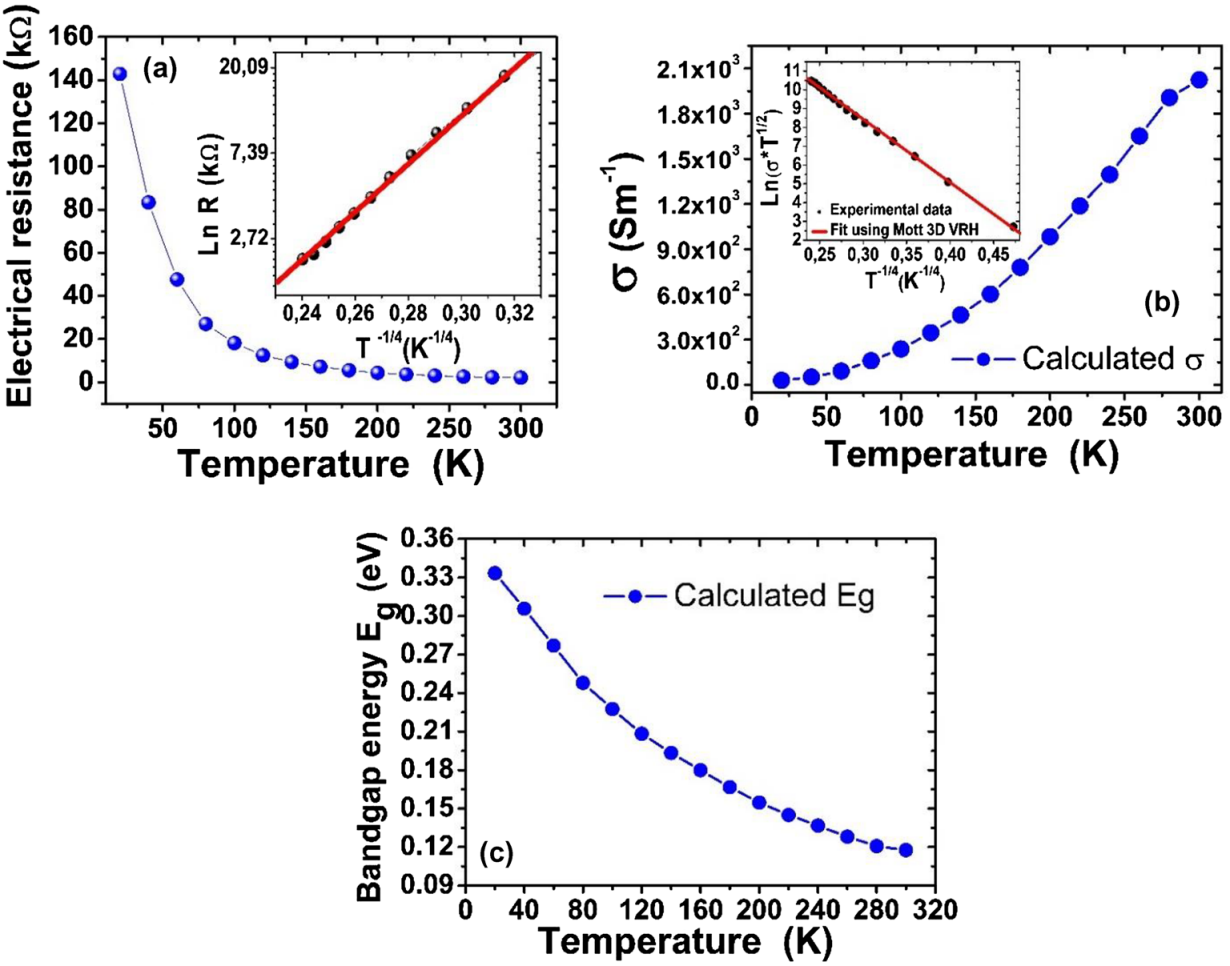
Variation of (**a**) electrical resistance, (**b**) electrical conductivity with temperature. Inset: Theoretical fit employing Mott's 3D-VRH model from expression (1). (**c**) Variation of bandgap energy with temperature in the GOF photodetector proposed here.

From the I–V curves analysis, it was possible to determine electrical resistance, electrical conductivity, and bandgap energy. Figure 5a shows the temperature dependence on electrical resistance for the photodetector prototype. It was found in all GOF photodetectors that as temperature decreases, electrical resistance increases, as expected for semiconductor materials^61,62^. The behavior with the temperature of the electrical resistance presented in Fig. 5a, was determined from the slope of the current–voltage curves, by using a linear fit to each experimental data at different temperatures. The electrical resistance values determined were 142.00 kΩ at the lowest temperature and 3.78 kΩ at room temperature, measurements at higher temperatures (above 300 K) will be reported in future works. There were some differences on the values of this parameter for all the GOF devices, which could be associated with the GOF fabrication processes, considering variations in the sizes of the GO samples and differences in the distances between the electrical contacts. It was also observed that as temperature decreases, the electrical resistance of the GOF prototypes increases, as expected in a semiconductor material, as reported by Muchharla^45^ et al*.*, and Babaev et al.^66^. The inset in Fig. 5a shows Ln (R) versus T^-1/4^ and the theoretical fit was best described employing the 3D-VRH model (Eq. (2)). A high concordance between the theoretical and experimental values can be observed. From the respective analysis, it was found that the linearity factor is 0.993, the characteristic temperature T_0_ exhibited values around 6 × 10^5^ K for the photodetectors developed in this work, which agree in order of magnitude with the range of values from 2 × 10^3^ to 8 × 10^5^ K reported for rGO in references^38,44^.

Temperature dependence on electrical conductivity is presented in Fig. 5b. The calculation of conductivity values was made from the electrical resistance value and geometric parameters, and it can be observed in the Fig. 5b a non-linear behavior with temperature, as expected for a semiconductor material. Increased temperature from 20 to 300 K, resulted in a conductivity rise by two orders of magnitude, starting from 3.0 × 10^1^ S m^-1^ and reaching a value of 2.02 × 10^3^ S m^-1^, agreeing very well with the results reported by K. Gross et al.^46^ and other reports in the literature on rGO obtained through chemical/thermal reduction methods^10^. The inset in Fig. 5b is the plot of ln(σT^1/2^) as a function of T^−1/4^ for all the range of temperatures considered, along with the best fitting line shape from the 3D-VRH model from expression (1). It is observed a good linear behavior with a linearity factor of 0.995, and from the slope, *T*_0_ was determined in 6.0 × 10^5^ K, employing σ_0_ = 2.0 × 10^4^ S m^-1^, as the reported value for graphite at room temperature^46^. These results revealed that GOF samples exhibited semiconductor behavior attributed to the 3D-VRH mechanism, as expected. The changes on the bandgap energy with the temperature are shown in Fig. 5c and the values were calculated by using the relationship between σ and E_g_ given by expression (2). Bandgap energy shows a nonlinear variation from 0.117 eV at 20 K to 0.333 eV at 300 K, which matches the reported range for rGO^65,67,68^.

### Optical characterization

**Figure 6:**
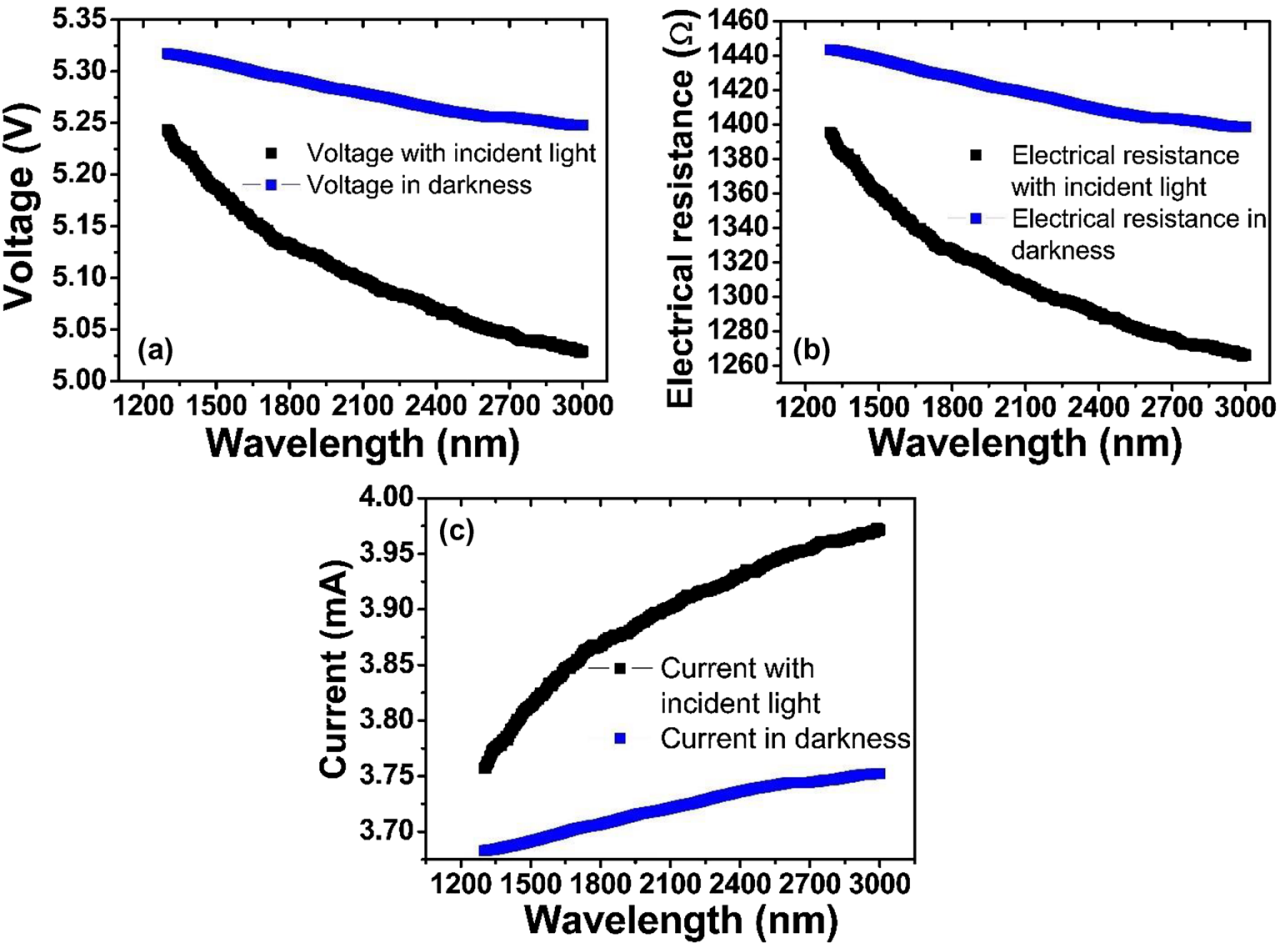
(**a**) Voltage vs. wavelength for the device. (**b**) Resistance vs. wavelength for the device. (**c**) Current vs. wavelength for the device.

Figures 6a–c show the dependence on voltage, electrical resistance and current as a function of wavelength in the GOF photodetector proposed here. Black and blue curves represent the voltage, electrical resistance and current measured with the incident light beam on the sample surface and the measurement performed in darkness, respectively. Figures 6a, b demonstrate that as the excitation wavelength of the incident light beam increases, the value of the voltage measured in the photodetector prototype decreases non-linearly, possibly due to a greater contribution from the dispersive processes of the electron–hole pairs in the material, as reported in photodetectors based on PbI_2_ with graphene oxide doping by Sharma et al.^69^. Also, the voltage measured in darkness is higher than the voltage in the presence of incident radiation. This behavior could be attributed to the high electrical resistance exhibited by the photodetector at room temperature. Figure 6b shows the effect of the wavelength of the incident light beam on electrical resistance for the device, and the electrical resistance in darkness. The lower electrical resistance values observed when the GOF are under the incidence of the light beam is possibly attributed to the excitation of a greater number of carriers on the surface of the GO sample by the incident electromagnetic radiation and remain available for electrical current.

The electrical current in GOF device was measured indirectly by employing Ohm's law from the analysis of the circuit presented in Fig. 2a. The electrical current of the photodetector, as a function of the wavelength, is shown in Fig. 6c, by considering the incidence of light and darkness conditions. This confirms that the current in the presence of incident radiation is greater than in darkness conditions, which can be explained from the generation of electron–hole pairs, which can be dissociated by the external applied electric field. Variations of the electrical current in the order of μA were found when varying the wavelength of the incident light beam. It is noted that as the wavelength increases, the photodetector current increases for the incidence of the light beam, as expected in photodetectors with electrical photoconduction effect^69,70^.

**Figure 7:**
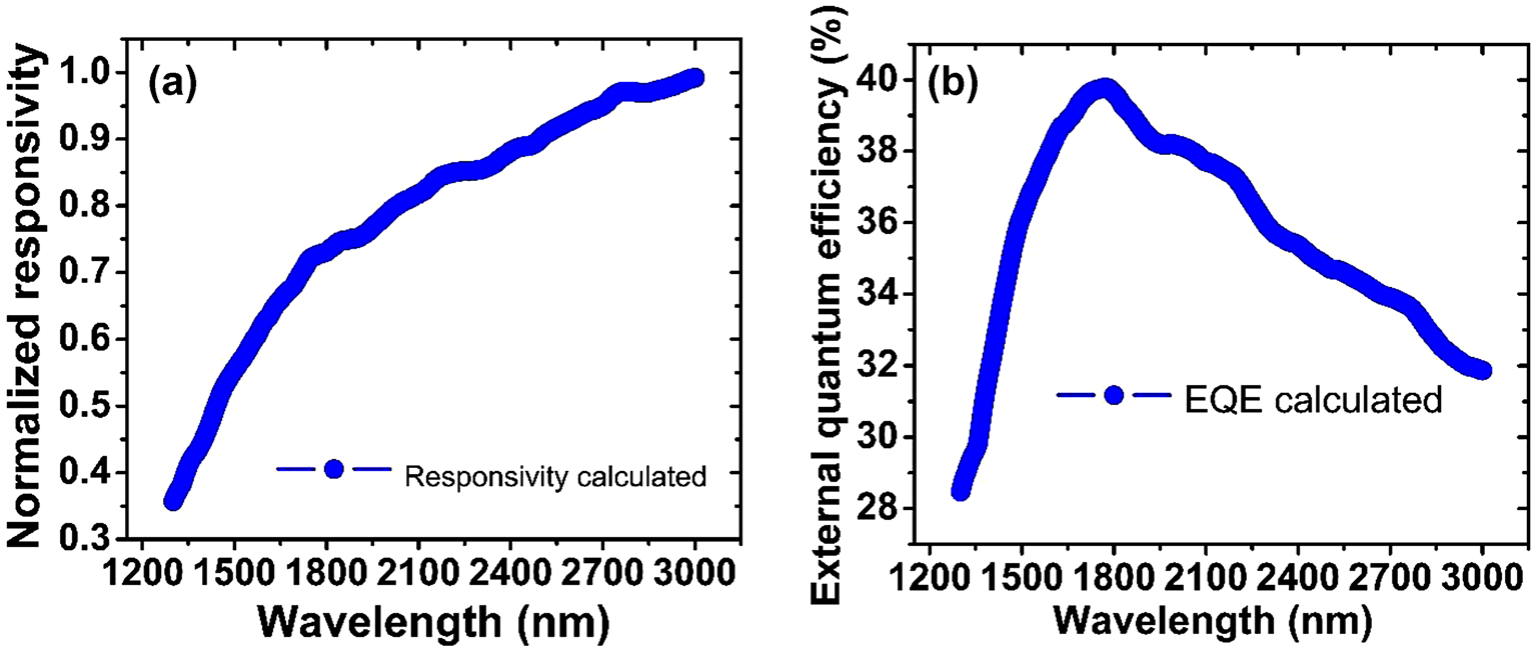
(**a**) Responsivity vs. wavelength for the device. (**b**) Quantum external efficiency vs. wavelength for the device.

Figure 7a presents the normalized value of responsivity as a function of wavelength. An important non-linear dependence on the responsiveness with wavelength can be observed in the GOF infrared photodetector, considering that they can generate a greater number of free electrons from the incident photons, as the wavelength increases. These results revealed a fine and systematic optoelectronic response of GOF infrared photodetector, and these are considered first measurements which can be used as an important reference to develop future infrared photodetector based on rGO with high EQE, as observed in Fig. 7b.

The external quantum efficiency was calculated employing Eq. (4) and plotted in Fig. 7b; for this, the electrical current values with incidence of light and in darkness conditions were considered to find the gain and responsivity values were calculated. It can be observed in Fig. 7b that, the photodetector can reach a maximum EQE value of 40%, which could be associated to the fact that the GOF photodetector prototypes were not encapsulated and, thus, the electrical contacts could undergo oxidation processes and furthermore, because the samples are exposed to ambient temperature and pressure, they may present other types of contamination. The high EQE value of 40% estimated here agrees with the EQE value of 41.81%, as reported by Alam^71^ et al. Additionally, in the near infrared spectral range, the EQE starts with low values due to the surface recombination of electron–hole pairs in the GOF material^72,73^. The EQE maximum value is reached because more free electrons are generated, but then it decays due to reflections and the low diffusion length of free electrons, as expected in commercial photodetectors^72,73^. Also, the EQE vs wavelength in Fig. 7b exhibits an optical response similar to the characteristic line shape in a Vis–NIR photodetector based on graphene^70^. The EQE behavior presented in Fig. 7b for the GOF device elaborated in this work agrees well, as expected for commercial photodetectors, and constitutes an important result in this work, given that it contributes to the concept that in the near infrared spectral range, GOF only exhibit the surface effect on which the light beam falls and do not show effects of the material’s volume, due to surface electrical contact; this behavior could be attributed to the low length of the light beam, compared to the 6 μm thickness of the GO sample.

## Conclusions

Pyrolytic GO samples were prepared using the double thermal decomposition method from the precursor Guadua *angustifolia Kunth* at 973 K, under a controlled nitrogen atmosphere. Photodetector prototypes were proposed here employing the GOF samples and exhibited a high external quantum efficiency of 40%, similar to those reported for commercial photodetectors. Electrical characterization varying temperature revealed that GOF exhibits a narrow bandgap semiconductor behavior, and it was found that the dominant electrical transport mechanism can be attributed to Mott's three-dimensional variable range hopping. In the optical characterization, measurements of voltage and electrical resistance, as a function of wavelength were considered here, and it was found that increased wavelength increases responsivity. It was evidenced that as the wavelength becomes longer, a greater number of free electrons are generated, which contribute to the electrical current. In addition, the electrical current obtained was greater in the presence of incident light radiation than in the dark, since the incident radiation stimulates a greater number of electron–hole pairs in the material. These results suggest GOF as an excellent candidate material to develop highly efficient IR photodetectors with 3D-VRH as the main electrical transport mechanism.

## Data Availability

The datasets used and/or analyzed during the current study available from the corresponding author on reasonable request.
